# Complementary and alternative medical therapies for chronic low back pain: What treatments are patients willing to try?

**DOI:** 10.1186/1472-6882-4-9

**Published:** 2004-07-19

**Authors:** Karen J Sherman, Daniel C Cherkin, Maureen T Connelly, Janet Erro, Jacqueline B Savetsky, Roger B Davis, David M Eisenberg

**Affiliations:** 1 Center for Health Studies, Group Health Cooperative, Seattle, Washington 98101, USA; 2 Department of Epidemiology, University of Washington, Seattle, Washington 98195, USA; 3 Departments of Family Medicine and Health Services, University of Washington, Seattle, Washington 98195, USA; 4 Department of Ambulatory Care and Prevention, Harvard Medical School and Harvard Pilgrim Health Care and Harvard Vanguard Medical Associates, Boston, Massachusetts 02215, USA; 5 Harvard Medical School Osher Institute and Division for Research and Education in Complementary and Integrative Medical Therapies, Harvard Medical School, Boston, Massachusetts 02215, USA; 6 Beth Israel Deaconness Medical Center, Boston, Massachusetts 02215, USA

**Keywords:** acupuncture, chiropractic, massage, meditation, t'ai chi, low back pain

## Abstract

**Background:**

Although back pain is the most common reason patients use complementary and alternative medical (CAM) therapies, little is known about the willingness of primary care back pain patients to try these therapies. As part of an effort to refine recruitment strategies for clinical trials, we sought to determine if back pain patients are willing to try acupuncture, chiropractic, massage, meditation, and t'ai chi and to learn about their knowledge of, experience with, and perceptions about each of these therapies.

**Methods:**

We identified English-speaking patients with diagnoses consistent with chronic low back pain using automated visit data from one health care organization in Boston and another in Seattle. We were able to confirm the eligibility status (i.e., current low back pain that had lasted at least 3 months) of 70% of the patients with such diagnoses and all eligible respondents were interviewed.

**Results:**

Except for chiropractic, knowledge about these therapies was low. Chiropractic and massage had been used by the largest fractions of respondents (54% and 38%, respectively), mostly for back pain (45% and 24%, respectively). Among prior users of specific CAM therapies for back pain, massage was rated most helpful. Users of chiropractic reported treatment-related "significant discomfort, pain or harm" more often (23%) than users of other therapies (5–16%). Respondents expected massage would be most helpful (median of 7 on a 0 to 10 scale) and meditation least helpful (median of 3) in relieving their current pain. Most respondents indicated they would be "very likely" to try acupuncture, massage, or chiropractic for their back pain if they did not have to pay out of pocket and their physician thought it was a reasonable treatment option.

**Conclusions:**

Most patients with chronic back pain in our sample were interested in trying therapeutic options that lie outside the conventional medical spectrum. This highlights the need for additional studies evaluating their effectiveness and suggests that researchers conducting clinical trials of these therapies may not have difficulties recruiting patients.

## Background

Back pain is one of the most common and costly health problems in developed countries, where more than half of adults suffer from this condition each year [1] and 70% to 80% suffer from it at some time in their lives [2]. Patients with back pain are often dissatisfied with standard medical care [3], especially in comparison to care provided by alternative providers [4-6]. In fact, back/neck pain is the number one condition for which Americans seek complementary or alternative medical (CAM) care. During 1997, 30% of Americans with back problems visited CAM practitioners, especially chiropractors and massage therapists, for this condition and another 18% used CAM self-care [7]. Yet, to date, few of these therapies have been adequately evaluated for effectiveness, in part because of methodological challenges including recruiting sufficient numbers of participants, designing reasonable interventions and selecting appropriate control groups.

Prior to designing and conducting two pilot clinical trials evaluating the effectiveness of five different CAM therapies for chronic low back pain (LBP) in older (65+ years) and younger (20 through 64 years) adults, we sought to refine recruitment strategies. As part of this effort, we surveyed chronic low back pain patients about their interest in trying each of these five CAM therapies if included in their health plan benefits and as part of two separate clinical trials. In this descriptive and exploratory study, we also collected information about their knowledge of, experience with, and perceptions of each of these therapies.

## Methods

### Study design

From April to October 2001, we conducted telephone interviews with 249 patients who were currently suffering from non-specific low back pain that had persisted at least three months. The patients were members of a non-profit managed health care system (Group Health Cooperative in the Puget Sound region of Washington State) and a large multi-specialty group practice (Harvard Vanguard Medical Associates, Boston, Massachusetts). Our goal was to interview 150 patients from Group Health and 100 patients from Harvard Vanguard who were otherwise healthy and spoke English, with 50% of the interviews from adults 65 years or older. The Institutional Review Boards of Group Health Cooperative, Seattle WA and Harvard Pilgrim Health Care, Boston, MA approved the study.

### Sample

Using automated visit data, we identified and mailed letters to 787 patients with working phone numbers who visited a Group Health (n = 422) or a Harvard Vanguard (n = 365) primary care provider and had a diagnosis consistent with non-specific low back pain between 12 and 52 weeks previously. Because we were planning to use the results of the survey to help us refine recruitment strategies for two pilot randomized trials, our exclusion criteria for this study were those we planned to use in the subsequent trials. We therefore used the automated visit data to exclude the following individuals from our sample:

• those whose back pain may have been due to a specific disease or condition (i.e., sciatica, herniated disc, spondylolisthesis, spine fracture, vertebral fracture, cancer);

• those who had other pain conditions that could complicate the interpretation of trial results (rheumatoid arthritis, ankylosing spondylitis, fibromyalgia);

• those who were inappropriate candidates for one or more of the clinical trial treatments (aneurism, coagulation disorders, osteoporosis) or who were unlikely to be able to give informed consent or to participate in the baseline and follow-up assessments of the trial (Alzheimers disease, dementia, major psychoses, blindness, deafness).

Before administering the in-person survey, we asked eight screening questions and then excluded individuals who did not have back pain at the time of the interview or who had not had back pain for at least 12 weeks, who had previously had low back surgery, who reported having fractured a vertebrae, who were pregnant, who were involved in back-pain related litigation, who had serious health problems, or who could not speak English.

We tried to contact all mailees, but could not reach 28 (7%) persons from Group Health and 73 (20%) from Harvard Vanguard despite at least seven phone calls. Among the 394 Group Health and 292 Harvard Vanguard patients who were contacted, 57 from Group Health and 81 from Harvard Vanguard refused the interview and we could not determine their eligibility status, 195 from GHC and 104 from Harvard Vanguard were ineligible upon screening, and 142 from Group Health and 107 from Harvard Vanguard were eligible and interviewed. Thus, we were able to screen 70% of all mailees for eligibility (80% from Group Health and 58% from Harvard Vanguard). All screened and eligible mailees were interviewed.

In both areas, individuals aged 65 and older were more likely to refuse screening (in Seattle: 19% of 210 older adults vs. 8% of 212 younger; in Boston: 31% of 184 older adults vs. 13% of 181 younger) and those under 65 were less likely to be contacted by phone (in Seattle: 12% younger vs. 1% older could not be contacted; in Boston: 31% younger vs. 9% older could not be contacted).

Most patients were ineligible because they were not experiencing back pain at the time of the interview (n = 82 from Group Health and n = 68 from Harvard Vanguard) or their pain had not persisted for three months (n = 39 from Group Health and n = 11 from Harvard Vanguard).

### Survey questionnaire

We conducted a phone interview that lasted an average of 17.7 minutes (SD = 6.1; range = 8 to 50 minutes). It included questions about demographic characteristics (age, race/ethnicity, education); back pain characteristics (years since first episode of back pain lasting more than two weeks, number of days of pain in the last six months, bothersomeness of pain on a 0 to 10 scale, expectations of pain level one year from the time of interview, and use of medications in the past week); self-reported knowledge (measured on a five point scale) of five CAM treatments or self-care methods (acupuncture, chiropractic, massage, meditation, t'ai chi); previous use of these therapies for any reason and for back pain specifically (and helpfulness of the therapy for back pain relief); perceived harm from previous use of these therapies; expectations of helpfulness of these therapies for current back pain; willingness to try these therapies if offered by the health plan for no additional cost and for a $10 per visit co-pay; willingness to participate in two hypothetical clinical trials, one evaluating acupuncture, chiropractic, and massage and another involving massage, meditation, and t'ai chi. (Respondents were told that the control group in both trials would receive a book about self-management of back pain.) Finally, respondents were asked about which treatment they most preferred among those offered in each trial. Gender and geographic location were obtained for respondents from the enrollment files of each healthplan. General definitions of each therapy were provided only for respondents who informed the interviewers they did not know what a particular therapy was. Acupuncture was described as a system of healing that involved inserting hair thin needles into acupuncture points just beneath the skin or using other methods, such as heat, to stimulate these points, whereas chiropractic was defined as a system of therapy that uses manipulation to adjusts the spine and other body parts to "promote normal nerve functions". Massage was described as the systematic rubbing and manipulation of muscle and other tissues to relieve bodily infirmities, while meditation was defined as a "self-directed practice for relaxing the body and calming the mind". Finally, tai chi was described as a Chinese martial art that uses slow and smooth body movements and is often practiced for its purported health benefits. The survey was pre-tested on a convenience sample of 15 people (both older and younger) in Seattle and 5 people in Boston.

### Statistical analyses

We analyzed the data using the SAS statistical software version 6.12 (SAS Institute, Cary, NC). Descriptive data were characterized using percentages or medians. For each of the five CAM therapies, we performed separate exploratory logistic regressions to identify specific characteristics associated with 1) high degree of knowledge (4 or 5 on a 5-point scale) (five separate models), 2) prior use (for any reason and for back pain) (10 separate models), 3) high expectations of helpfulness for current back pain (7 to 10 on a 0 to 10 scale) (five separate models), 4) greatest likelihood of trying therapy for no additional cost (all five therapies) and for a $10 per visit co-pay (acupuncture, chiropractic, massage only) (eight separate models), and 6) greatest likelihood of participating in each of the two hypothetical clinical trials (two separate models). Thus, a total of 30 separate logistic models were created.

For each of the 30 dependent variables, we identified potential predictor variables in advance and evaluated them in preliminary models. In Table 1, the potential predictor variables evaluated in the preliminary models for each of the 28 therapy-specific dependent variables are indicated by an X. In addition, we modeled the likelihood of being "definitely willing" to participate in a hypothetical clinical trial of acupuncture, chiropractic, and massage and of being "definitely willing" to participate in a hypothetical clinical trial of massage, meditation, and t'ai chi. In both models, we evaluated the following 22 variables as potential predictor variables of being "definitely willing" to participate in the hypothetical clinical trial: demographic characteristics (age, gender, race, education, geographic location), prior use of each of the therapies included in the trial (i.e., acupuncture, chiropractic, and massage for one trial and massage, meditation, and t'ai chi for the other trial) for any reason (and for back pain), knowledge of these three included therapies, prior perceived harm from these three included therapies, years since first back pain, symptom bothersomeness, high expectations of each included CAM therapy for current back pain, number of days of back pain in last six months, and medication usage in the week prior to the interview.

**Table 1:** Potential Predictor Variables Evaluated in 28 Therapy-Specific Logistic Regression Models

	Dependent Variables for Logistic Regressions					
Potential Predictor Variable	High Knowledge of Therapy*	Prior Use of Therapy*	Prior Use of Therapy for Back Pain*	High Expectations of Success of Therapy*	Likelihood of Trying Therapy at No Cost*	Likelihood of Trying Therapy for $10 Co-pay**

Geographic location (Boston vs. Seattle)	X^¶^	X	X	X	X	X
Age (65+ vs. < 65)	X	X	X	X	X	X
Gender (female vs. male)	X	X	X	X	X	X
Race (white, non-white)	X	X	X	X	X	X
Education (no college vs. some college)	X	X	X	X	X	X
≥ 5 years since first back pain	X				X	X
≥ 90 days of LBP in last 6 mo.	X				X	X
High symptom bothersomeness (7 – 10) on a 0 – 10 scale	X				X	X
High knowledge of therapy (4 or 5) on a 1 – 5 scale				X	X	X
Prior use of therapy				X	X	X
Prior use of therapy for back pain				X	X	X
High expectations of therapy (7 – 10) on a 0 – 10 scale					X	X
Medication usage in past week					X	X
Prior harm from therapy					X	X

* Separate models were done for each of the five therapies (acupuncture, chiropractic, massage, meditation, t'ai chi) ** Separate models were done for acupuncture, chiropractic, and massage. ^¶^An X indicates that a particular potential predictor variable was evaluated in a model with the specific dependent variable.

Initially, we evaluated potential predictor variables in preliminary models containing five or fewer independent variables. Any independent variable associated with the dependent variable at a p value of 0.15 or less in a preliminary model was a candidate for the appropriate final model. We used a backwards elimination procedure to evaluate candidate predictor variables and to determine the final models [8]. All variables with a p value of 0.01 or less were retained in the final model. Odds ratios (OR) are presented along with 95% confidence intervals (95% CI). Table 4 presents the odds ratios that describe the significant associations (p < 0.01) for each of the 28 therapy – specific dependent variables.

**Table 4:** Predictors of Knowledge of, Experience with, Expectations about, and Willingness to Try Five Complementary and Alternative Medical (CAM) Therapies

	Odds ratios* (95% CI) for the independent predictor variables used in the final models for each CAM therapy				
**Dependent Variable**	**Acupuncture**	**Chiropractic**	**Massage**	**Meditation**	**T'ai chi**
High Knowledge of specific therapy (4–5)	Tried acupuncture: 43.6 (16.7–113.6)	Tried chiropractic: 12.8 (6.2–26.7)	Tried massage: 7.6 (4.0 – 14.7)	Tried meditation: 11.6 (4.6–29.3)	LOGISTIC NOT VALID**
				Bostonian: 4.8 (1.9–12.3)	
Previously tried specific therapy	No associations	Bostonian: 0.5 (0.3 – 0.8)	65+ yrs: 0.4 (0.2 – 0.7)	Female: 2.5 (1.3 – 4.8)	No associations
Previously tried specific therapy for back pain	No associations	None	65+ yrs: 0.3 (0.2 – 0.6)	No associations	LOGISTIC NOT VALID**
High expectations of specific therapy (7 – 10)	65+ yrs: 0.4(0.2 – 0.8)	Knowledge: 2.9 (1.6 – 5.2)	65+ yrs: 0.3 (0.2 – 0.5)	No associations	No associations
	Tried acupuncture: 4.3 (2.1 – 9.0)				
Very likely to try specific therapy for free	High expectations: 15.4 (3.6 – 66.1)	High expectations: 27.4 (9.5 – 79.3)	High expectations: 16.4 (7.4 – 36.5)	High expectations: 3.6 (1.7 – 7.7)	High expectations: 14.3 (5.4 – 38.3)
				Tried meditation: 2.4 (1.3 – 4.5)	
Very likely to try specific therapy for $10 /visit co-pay	High expectations: 6.8 (3.0 – 15.5)	High expectations: 8.1 (4.2 – 15.7)	High expectations: 6.4 (3.5 – 11.4)	NOT QUERIED	NOT QUERIED
	Bostonian: 2.3 (1.4 – 4.0)		Bostonian: 1.8 (1.001 – 3.2)		

*These odds ratios describe the significant associations (p < 0.01) for each of the 28 therapy – specific dependent variables. For example, we found that those who had tried acupuncture were 43.6 times more likely to have high knowledge of acupuncture. No other variables were related to high knowledge of acupuncture. ** These logistic regression models did not converge. Categorization for independent variables: Age (<65; 65+) Knowledge of therapy (1–3; 4–5) Gender (M; F) Expectations of therapy (missing through 6; 7+) Geography (Seattle; Boston) Prior Use of therapy (no; yes)

## Results

### Characteristics of respondents

Most study participants were white, women, and had attended college (Table 2). Most had had back problems for at least five years, had experienced back pain at least 90 days in last six months, had used medications in the prior week, and expected little change in their pain in a year.

**Table 2:** Demographic and Back Pain Characteristics of 249 Survey Respondents

Characteristic	Percent
Location (Boston)	43
Age (< 65)	52
Women	60
White	80
Attended some college	57
At least 5 years since first back pain lasting longer than 2 weeks	60
90+ days of LBP in last 6 mo.	61
High symptom bothersomeness in the past week (≥ 7) on 0 – 10 scale	42
Used medication for LBP in the past week	56
Expect pain to be similar in a year	72

Missing data – last variable has 10 missing values (4% of all observations), 1 variable has 5 (2%), all others have 3 or fewer.

### Knowledge of, experience with, perceptions of, and willingness to try CAM therapies

Except for chiropractic, most participants reported little or no knowledge of these therapies (Table 3). In logistic regressions, prior use of a therapy consistently predicted high knowledge of that therapy (Table 4).

**Table 3:** Knowledge of, Experience with, Expectations about, and Willingness to Try Five CAM Therapies*

	Acupuncture (N = 249)	Chiropractic (N = 249)	Massage (N = 249)	Meditation (N = 249)	T'ai Chi (N = 249)
Knowledge about Therapy (%)					
1 – 2 (1="no knowledge")	69	44	52	72	91
3	17	22	24	15	6
4 – 5 (5="a lot of knowledge")	14	34	24	13	3
Ever tried therapy (%)	18	54	38	27	8
Ever tried therapy for LBP (%)	11	45	24	7	0.4
Median helpfulness for LBP among prior users (0 to 10 scale)	5	6	7	5	**
Pain or harm reported by prior users (%)	13	23	13	5	16
Median expectation of helpfulness for current LBP (0 to 10 scale)	5	5	7	3	5
Did not provide expectation rating (%)	25	10	9	12	24
High expectations of helpfulness for current LBP (7 to 10 on 0 to 10 scale) (%)	19	28	48	15	16
Very likely to try therapy if primary care provider thought reasonable and no extra cost (%)	64	51	69	27	41
Very likely to try therapy if primary care provider thought reasonable and $10 co-pay (%)	51	42	56	NA	NA

NA = Not Asked. * Each column refers to a specific therapy and the specific question about the therapy is shown in the first column. ** Only 1 person had tried t'ai chi for low back pain previously. All variables, except expectations of helpfulness of current LBP (where % are given in the table) have missing values for < 5% of respondents.

More than half of the participants had tried chiropractic compared with 38% who had tried massage and substantially fewer who had tried the other therapies (Table 3). No demographic characteristics were related consistently to use of these therapies (Table 4). Chiropractic and massage were also the most commonly used of the therapies specifically for low back pain. Users of massage rated treatment helpfulness higher than did users of other therapies (Table 3). Reports of harm or increased pain were highest for chiropractic (23%) and lowest for meditation (5%).

Respondents believed that massage would be most helpful for their current back pain (median rating of 7) and that meditation would be least helpful (median rating of 3) (Table 3). One quarter of all respondents were unable to rate their expectation of acupuncture or t'ai chi, compared to about 10% for the other therapies. Respondents 65 years of age or older were less optimistic than younger respondents about the helpfulness of acupuncture and massage (Table 4). High expectations of helpfulness of chiropractic were more common in those with high knowledge of this therapy and high expectations of helpfulness of acupuncture were more common among those who had tried it (Table 4).

More than half of the respondents said they would be "very likely" to try acupuncture, chiropractic, or massage if provided by their health plan for no additional cost and their physician felt it was reasonable. Fewer respondents said they would be "very likely" to try meditation training (27%) or t'ai chi (41%) under those circumstances (Table 3). In logistic regression models, the strongest predictors of being very likely to try a particular therapy were high expectations of a therapy and, for meditation, prior use of the therapy (Table 4). About 80% of those very likely to try acupuncture, chiropractic, or massage for no additional cost were also very likely to try it for a $10 per visit co-pay (Table 3). Paralleling the finding for free care, the strongest predictor of willingness to try a therapy for a $10 per visit co-pay was high expectations of success for that therapy. Respondents from Boston were more willing to try acupuncture. Those reporting harm or pain from chiropractic were less willing to try this therapy again.

### Willingness to participate in a clinical trial

More than half of respondents were "definitely willing" to participate in each of two hypothetical clinical trials about which they were asked and less than 5% were definitely unwilling to participate (Table 5). When asked which of the treatments in each trial they would most prefer, respondents preferred massage and acupuncture in the trial of acupuncture, chiropractic, and massage and, in the second trial, strongly preferred massage to meditation. However, a significant fraction (24%) expressed a preference for t'ai chi. We found no demographic, back pain, or CAM characteristics associated with being "definitely willing" to participate in the hypothetical trial of acupuncture, chiropractic, and massage. People who were "definitely willing" to participate in the hypothetical trial of massage, meditation, and t'ai chi were more likely to have high expectations of meditation (OR = 3.1, 95% CI = 1.4 – 7.0).

**Table 5:** Willingness to Participate in Clinical Trials of CAM Therapies for Low Back Pain and Preference for Therapies

	Percent (N = 249)
Definitely willing to participate in clinical trial of acupuncture, chiropractic, massage, and a self-help back pain book (%)*	62
Preferred treatment among above:	
Massage	43
Acupuncture	35
Chiropractic	18
None or Other	3
Book	1
Definitely willing to participate in clinical trial of massage, meditation, t'ai chi, and a self-help back pain book (%)**	53
Preferred treatment among above:	
Massage	63
T'ai Chi training	24
Book	5
Meditation training	4
None or Other	4

Missing values – < 4% of responses for each variable are missing. *Your healthplan is thinking about conducting a study evaluating several treatments for people with chronic low back pain. In this study, participants would have a one in four chance of being assigned to one of the following treatments: acupuncture, chiropractic, massage, or a book designed to help patients better understand their low back pain. Participants would be expected to try the treatment they were assigned to at least once. Participants would still retain access to their usual care and participation in this study would be free. If you were asked to take part in a study like this would you be willing to participate? ** Same question was asked, but the treatments were massage, meditation training, and t'ai chi training.

## Discussion

Our findings suggest that many patients would be willing to try specific CAM therapies for back pain, especially if they had high expectations for their helpfulness. Interestingly, we found no consistent relationships between high expectations for a particular therapy and either previous use of that therapy or high self-perceived knowledge of that therapy.

Our findings regarding knowledge, previous use, and expectations for these therapies were largely similar for Seattle and Boston and for older and younger adults. However, those over 65 years old were less likely to have high expectations of acupuncture and massage and to have tried massage previously.

Since we conducted the study in two metropolitan areas where CAM use is fairly common, our results might not represent the CAM views of patients with back pain in more rural areas or in other regions of the country. Another limitation of our study was that 30% of people we attempted to contact could not be assessed for eligibility, leading to the possibility of a high non-response rate. Because we have almost no information on the characteristics of the individuals with unknown eligibility, we do not know if they differ from those included in the study, and cannot adequately estimate the magnitude and direction of potential biases regarding interest in CAM that might exist in our sample. However, the fractions of individuals who were unable to be assessed for eligibility were similar among those less than 65 years of age and those 65 and older in each metropolitan area (45% vs. 40% in Boston, respectively; 20% vs. 20% in Seattle).

Respondents showed a clear preference for receiving hands-on treatments delivered by a practitioner compared to attending classes that teach self-care techniques. Whether this reflects a preference for provider-oriented, more passive, therapies or the belief that classes teaching these specific self-care therapies would be less effective is not clear. Unfortunately, our interview did not include questions about yoga, which has recently received more popular press than meditation or t'ai chi as a self-care therapy for back pain [9,10].

Survey respondents were not enthusiastic about "meditation training" as a treatment for back pain. Relatively few of those who indicated prior use of meditation for physical or mental health problems had used the forms of meditation most commonly taught in a medical setting (e.g., mindfulness meditation). Consequently, studies recruiting patients to participate in interventions including meditation training may need to carefully describe the treatment in terms of a concrete goal (e.g., stress reduction).

There is still relatively little knowledge about and experience with acupuncture and t'ai chi even in Boston and Seattle where use of CAM therapies is generally high. In fact, about one – quarter of respondents were unable to provide an expectation of the helpfulness of acupuncture or t'ai chi. Nevertheless, substantial fractions of participants were willing to try acupuncture and t'ai chi as a treatment if their primary care provider thought it reasonable, and in the case of acupuncture, even if they had to pay a $10 co-pay each visit. Our finding that people in our sample reported being almost as willing to try acupuncture as massage, despite less knowledge of, expectations about and experience with it, is intriguing and requires further inquiry.

Although participants in this study reported more knowledge of and experience with chiropractic, they were more enthusiastic about massage. A recent survey [11]of 46,000 Consumer Reports subscribers found that among those who had experienced back pain, the relatively few who had tried deep tissue massage rated it more favorably than those who had tried medications or physical therapy. The use of massage in this country has been growing steadily since the 1960's, with the largest increases in the 1990's [12]. In fact, in surveys of CAM use in the US population conducted in 1990 and 1997, Eisenberg et al. [7] found that massage as a treatment for various medical conditions had increased 61% over the seven-year period, while chiropractic remained fairly stable. By 1997, the estimated percentage of US adults who had used chiropractic was similar to that who had used massage, 11%. The relative popularity of massage may result from the more positive experiences of those who have tried it compared with chiropractic or acupuncture, and higher expectations that massage would be helpful for their current pain. Moreover, chiropractic users were more likely to report treatment related "harm" or "pain" than were users of massage.

### Implications for clinical trials

Most survey respondents indicated they were "very willing" to participate in our two hypothetical clinical trials evaluating different CAM treatments for chronic back pain. Massage was the preferred treatment in both trials, but more than one in five survey respondents stated a preference for acupuncture and t'ai chi. In view of the long-standing popularity of chiropractic, surprisingly few respondents reported chiropractic as their top choice. Nonetheless the finding that massage was substantially more popular than chiropractic mirrors the results among acute low back pain patients in a clinical trial who were randomized to a choice of acupuncture, chiropractic, massage, or usual care or to usual care alone [13]. In that study, 52% of the participants said they would choose massage if given a choice, compared with only 24% who said they would choose chiropractic if given a choice. This finding could reflect the fact that many people have access to chiropractic as part of their current health care coverage [14].

Despite low levels of knowledge about t'ai chi and acupuncture, the finding that over 40% of respondents indicated they were very likely to try these therapies suggests that recruiting enough subjects for clinical trials involving these therapies may be feasible if moderate to large patient populations are available. Recruiting patients for meditation trials, however, is likely to be difficult. Consequently, when we recruited patients for a pilot trial that included a stress reduction intervention based on the principles of mindfulness meditation, we chose to describe it as "Mindfulness Based Stress Reduction" rather than mindfulness meditation.

We believe that clinical trials evaluating obviously different treatments for chronic low back pain, such as massage and meditation, may have problems retaining subjects who do not receive the treatment (e.g., massage) that attracted them to the study. This problem may be exacerbated if patients have an exceptionally strong preference (or dislike) for one treatment. Inclusion of multiple CAM modalities in a single study risks tempting potential participants to sign up for the study in the hope of receiving a desired treatment, and then dropping out if they receive a different treatment.

In addition, if one treatment is vastly more popular than another, it could be difficult to disentangle the effects of patient expectations and treatment efficacy per se, leading to difficulties in interpreting positive study outcomes. This problem is compounded by concerns about the subjective nature of back pain outcomes, the difficulty in masking participants to study treatment, and the strong skepticism of some researchers that CAM treatments can be effective, even when results are impressive. Masking patients to treatment is quite difficult in studies of many types of conventional as well as CAM treatments if the treatments involve a physical modality, such as massage, or active participation of the patient in the treatment, as in t'ai chi. In such circumstances, using masked outcomes assessors is important to minimize bias. We also suggest that patient (and provider) expectations for treatment and prior experience with each treatment, be measured and, if appropriate, controlled for in the analyses. Finally, if a particular therapy is shown effective in clinical trials in different populations, mechanistic studies will be important for determining how these therapies achieve their effects. Such studies are especially important to convince skeptics that CAM therapies actually have specific effects. In the meantime, the high and rising public interest in CAM therapies, especially for musculoskeletal conditions [12], highlights the importance of evaluating the effectiveness of various CAM treatments for back pain and our findings suggest that recruiting for these efforts may not be difficult.

## Conclusions

Most patients in our sample were interested in trying options for treating chronic back pain that lie outside the conventional medical spectrum, even within the context of a clinical trial. This was true even among patients who had relatively little knowledge of or experience with the therapy. Given our limited knowledge about the effectiveness of most CAM therapies, there is a clear need for additional studies evaluating their effectiveness. Fortunately, our results suggest that researchers will not find it difficult to recruit patients interested in participating in clinical trials of many of the CAM therapies.

## Competing interests

None declared.

## Authors' contributions

KJS participated in the development of the questionnaire and took primary responsibility for the analysis of the data and for drafting the manuscript. DCC was the PI on one of the grants funding the study, participated in the development of the questionnaire and the analysis of the data and played a major role in drafting the manuscript. MTC participated in the development of the questionnaire and the analysis of the data. JE and JBS coordinated the project and oversaw the data collection. RBD participated in the analysis of the data and provided statistical oversight. DME was the Principal Investigator on one of the grants funding the study, participated in the development of the questionnaire and the analysis of the data. All authors read and approved the manuscript.

## Pre-publication history

The pre-publication history for this paper can be accessed here:


